# Preparation of Soft Magnetic Fe-Ni-Pb-B Alloy Nanoparticles by Room Temperature Solid-Solid Reaction

**DOI:** 10.1155/2013/946897

**Published:** 2013-11-20

**Authors:** Guo-Qing Zhong, Qin Zhong

**Affiliations:** State Key Laboratory Cultivation Base for Nonmetal Composite and Functional Materials, School of Material Science and Engineering, Southwest University of Science and Technology, Mianyang 621010, China

## Abstract

The Fe-Ni-Pb-B alloy nanoparticles was prepared by a solid-solid chemical reaction of ferric trichloride, nickel chloride, lead acetate, and potassium borohydride powders at room temperature. The research results of the ICP and thermal analysis indicate that the resultants are composed of iron, nickel, lead, boron, and PVP, and the component of the alloy is connected with the mole ratio of potassium borohydride and the metal salts. The TEM images show that the resultants are ultrafine and spherical particles, and the particle size is about a diameter of 25 nm. The largest saturation magnetization value of the 21.18 emu g^−1^ is obtained in the Fe-Ni-Pb-B alloy. The mechanism of the preparation reaction for the Fe-Ni-Pb-B multicomponent alloys is discussed.

## 1. Introduction

The various metals and alloys have been widely used in science and technology. Many amorphous alloys, such as Fe-based amorphous alloys, possess superior magnetic properties compared to their crystalline counterparts because of their unique atomic structures [1]. A great deal of effort has been devoted to iron series alloy nanoparticles because of the novel optical, catalytic, electrical, and magnetic properties [2–8]. Generally, the preparation methods of the nanoalloys have strict environment requirement or complicated experiment process because of the strong activity of the fresh metal nanoparticles [9–12]. The metal-boron alloys nanoparticles were generally prepared in solution by chemical reduction method. But a variety of synthetic parameters, such as the ratio of the reactants, pH of the solution, and the order of the materials added, have significant effect on the morphology of the obtained materials in the aqueous chemical reduction which has been researched in recent years [13–17]. Room temperature solid-solid chemical reaction is a novel technique for preparation of nanosized materials. Recently, we have found that some amorphous alloys nanoparticles and the copper, bismuth, or antimony nanocrystalline particles can be prepared very easily by room temperature solid-solid reaction [18–21]. The reaction can be quickly accomplished at room temperature, which is a challenge to those traditional methods.

Lead can form eutectic alloy [22, 23] or hypomonotectic alloy [24–26] with another metal and is applied to the battery or lubricating materials, electrical contact materials, superconducting materials, and so on. In recent years, the prepared method and character of a series of lead alloys have been studied. The preparation and characteristics of Fe-Pb, Ni-Pb, and a series of ternary alloys about lead have been reported [27–29]. Lead is a soft metal and it is insoluble in iron under equilibrium conditions. Therefore, the study on the room temperature solid-solid chemical reaction preparation of the lead-based multicomponent alloy nanomaterials will be very interesting not only for studying incompatibility alloy, but also for exploration of the new preparation method of other multicomponent alloy nanoparticles. In this work, the Fe-Ni-Pb-B multicomponent alloy nanoparticles are synthesized by room temperature solid-solid chemical reaction, the effect of the mole ratio of potassium borohydride and the metal salts on the component of the alloy nanoparticles is discussed, and the magnetic performance is tested.

## 2. Experimental

All chemicals used in the preparation experiment were in analytical grade, which were purchased from Chengdu Kelong Chemical Reagent Factory in China. Ferric trichloride hexahydrate, nickel chloride hexahydrate, and lead acetate trihydrate were weighed and placed in an agate mortar and mixed up, and the corresponding mole ratio of the three metal salts was 1.00 : 1.00 : 0.500. Then, the white potassium borohydride powder was added to the above mixture, and the mole ratio of the metal salts to potassium borohydride was 1.00 : 1.25. Immediately, the mixture became black and released a lot of colorless gas. The released gas was tested with moist pH paper, and the result indicated that the gas was faintly acid gas. The reason was that the acetic acid and hydrogen chloride were released in the reaction process. The mixture was grinded carefully about 3 min, and then 2% of polyvinylpyrrolidone (PVP) of the total mass of the metal salts was added to the above agate mortar, and the grinding was kept on about 5 min. The reaction was conducted at room temperature. Then, the resultant was moved to a glass beaker as soon as possible, and the resultant was rinsed repeatedly by deionized water and collected by a centrifugation for removal of the residual reactants until no chlorine ion in washed water could be tested by the silver nitrate solution. Afterwards, the resultant was washed thoroughly by ethanol to remove residual water in the resultant. Finally, the resultant was dried in vacuum at 313 K for 5 h. The resultant was fine black particles and recorded as **1**. The same method was used to prepare resultants **2** and** 3**, in which the mole ratio of the metal salts to potassium borohydride was 1.00 : 2.00 and 1.00 : 3.00, respectively.

**Figure 1:**
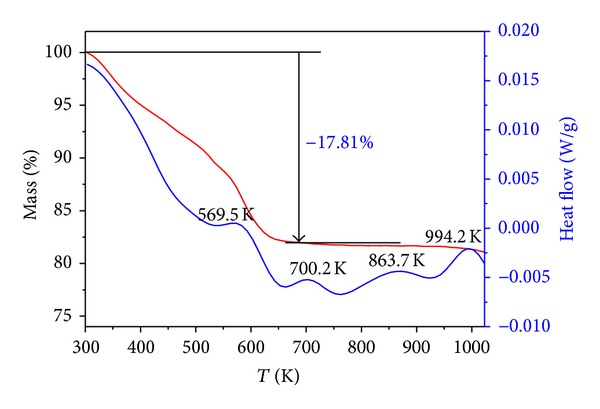
TG-DTA curves of resultant **2**.

**Figure 2:**
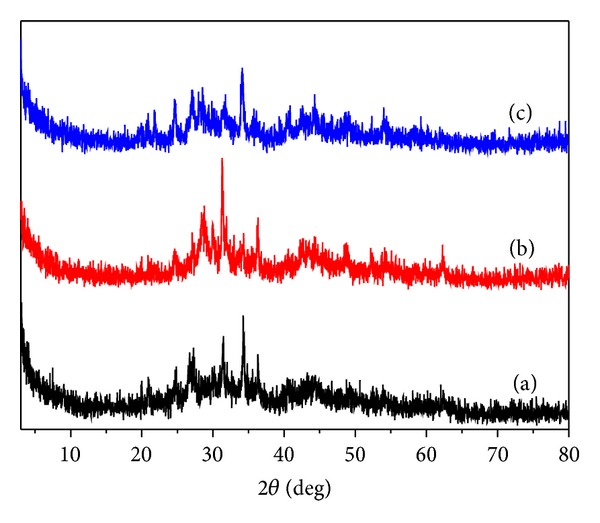
Powder X-ray diffraction patterns of the Fe-Ni-Pb-B alloy nanoparticles: (a) resultant **1**, (b) resultant **2**, and (c) resultant **3**.

**Figure 3:**
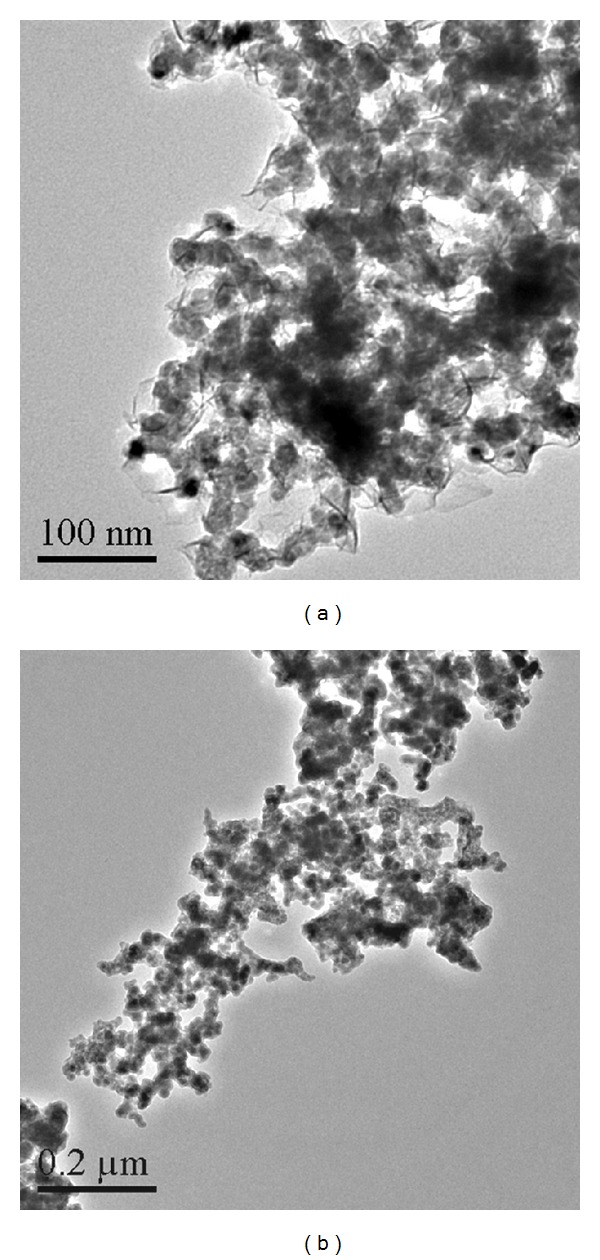
Transmission electron micrographs of the Fe-Ni-Pb-B alloy nanoparticles.

The composition of the resultants was determined by the inductively coupled plasma-atomic mass spectrometer. First, the sample was solubilized by a certain amount of concentrated nitric acid. Then, the content of the iron, nickel, lead, and boron in the sample solution was measured by a Thermo X-2 ICP-MS instrument. The thermal properties of the resultant were examined by a TA Q500 thermogravimetric analyzer at a heating rate of 20 K min^−1^, and the TG-DTA curves of the resultant were shown in Figure 1. The powder X-ray diffraction (XRD) patterns of the resultants were recorded by a *D*/max-II X-ray diffractometer, Cu K_*α*1_ radiation (*λ* = 0.154056 nm), and Ni filter, and scanning rate was 8° (2*θ*) min^−1^ at room temperature. The powder X-ray diffraction data of the resultants were collected in the diffraction angle ranges of 3°–80° in Figure 2. The morphology and the particle sizes of the resultant were studied by a Tecnai G20 (FEI) transmission electron microscopy at 300 kV. For TEM observation, the samples were dispersed in ethanol by ultrasonic treatment and dropped on carbon-copper grids. The TEM images of the resultant were given in Figure 3. The soft magnetic properties of the resultants were investigated by a BKT-4500Z vibrating sample magnetometer.

## 3. Results and Discussion

The chemical composition and the percentage content of the resultants are listed in Table 1. The results of the ICP analysis indicate that the percentage content of iron, nickel, lead, and boron in the resultant **2** is 9.66%, 26.45%, 46.50%, and 0.31%, respectively. The total content of the metals and boron of resultant **2** is about 82.92%. As the PVP was added, the total percentage content of the metals and boron is not equal to 100%. Therefore, the other elements (17.08%) in the resultant most possibly come from the PVP, and the conjecture is demonstrated in the result of TG-DSC analysis. In addition, several compositions of the alloy nanoparticles have been gained by adding a different ratio of KBH_4_ in the experimental process. Increasing the addition of potassium borohydride, from Table 1, the percentage content of iron and nickel in the resultant is increased. This indicates that the mole ratio of potassium borohydride to the metal salts has an obvious effect on the composition of the resultant.

The TG-DTA curves of resultant **2** in air from room temperature to 1023 K are shown in Figure 1. There is an obvious weight loss process in the TG curve below 650 K. Because the resultant contains iron, nickel, lead, boron, and PVP, so the lost component must come from the oxidation and decomposition of PVP. The total percentage weight loss is 17.81%, and it fits right in with the data (17.08%) of the ICP determination. The weight of the residue is constant at 650 K. There are multiple weak exothermic peaks in the DTA curve from 650 to 1023 K, which are caused by the crystallization of the resultant in heating process. The results of the ICP and thermal analysis indicate that the resultant is not oxidized. In fact, the fresh metal alloy particles can be oxidized very easily by the oxygen in the air [21]. In this case, the particles can be cladded by PVP to insulate oxygen from air.

By now, there is no literature that reported the preparation of the high lead multicomponent amorphous alloys. We observe that the sharp peaks and diffuse peak exist in the X-ray powder diffraction patterns in Figure 2. This indicates that the resultants are mainly consisted of nanoamorphous phase and a minute amount of nanocrystalline phase [2]. The probable reason is that the reactions are very quickly carried out at room temperature, and the generated particles cannot accomplish the conversion from amorphous state to crystalline state. At the same time, the self-diffusion of lead and the incompatibility alloy formed easily of lead and other metals may be the main reason [30]. Combined with the TEM images of resultant **2**, two different crystal states have been found in Figure 3. In addition to a large amount of spheroidal particles, some linear products are scattered in the resultant. This may explain the existence of sharp peaks and diffuse peak in the XRD patterns. A statistical analysis of the TEM images shows that the resultant is mainly formed from ultrafine, and spherical nanoparticles and a small amount of nanowires are interspersed among them. The average particle size of the spherical nanoparticles is measured from the TEM images and estimated to be of diameter 25 nm. A layer of film can be observed in Figure 3, that is the PVP which is coated on the surface of the nanoparticles, and it is the probable reason that the fresh metal alloy nanoparticles have not been oxidized easily.

**Figure 4:**
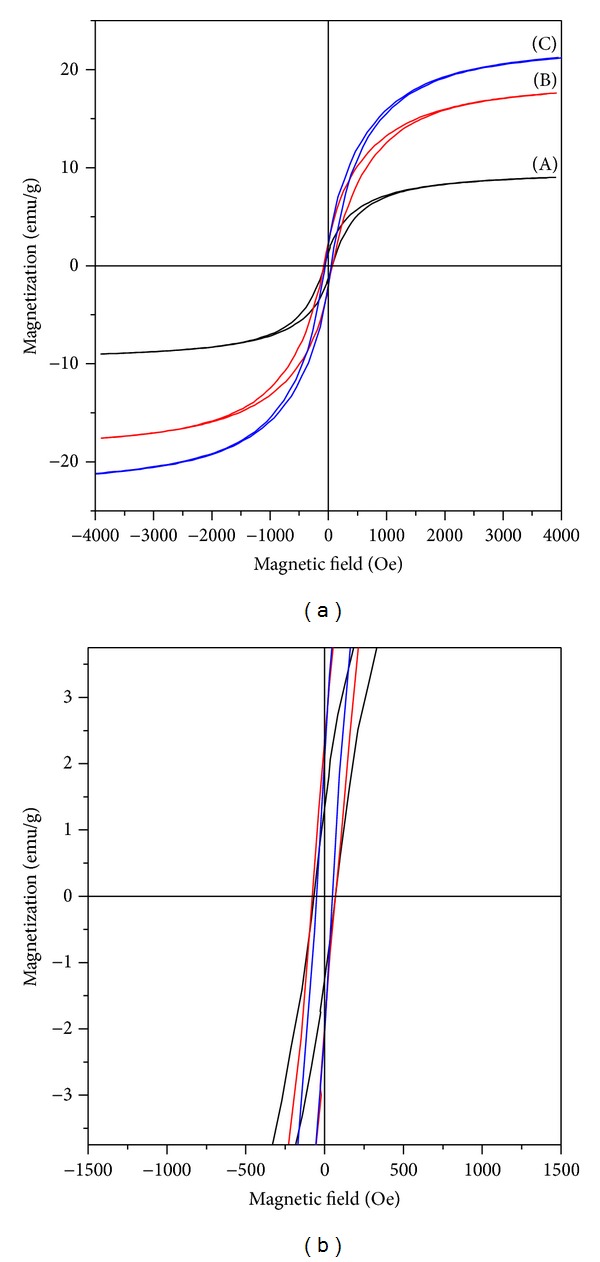
Complete magnetic hysteresis curve (a) and partial of this curve showing the coercive force (b) of the Fe-Ni-Pb-B alloy nanoparticles: (A) resultant **1**, (B) resultant **2**, and (C) resultant **3**.

Figure 4(a) illustrates the hysteresis loops of resultants **1**, **2**, and **3**, respectively. The data of saturation magnetization, coercivity, and remanent magnetization for resultants** 1**,** 2**, and **3** is listed in Table 2. It can be seen that the increase in the metallic relative content in resultants causes significant enhancement of the saturation magnetic polarization (*Ms*) from 9.01 emu g^−1^ for **1 **to 21.22 emu g^−1^ for **3**; namely, it is increased with reducing agent addition.The determined data of the coercive force (*Hc*) for **1**, **2**, and **3** is similar and is, respectively, 65.85, 77.85, and 54.17 Oe which are graphically illustrated in the corresponding Figure 4(b). The long and narrow hysteresis loops indicate that the resultants have low coercive force and remanent magnetization. Therefore, the resultants have superior soft magnetic property.

The preparation of multicomponent nanoalloys such as the Fe-Ni-Pb-B alloy nanoparticles by a room temperature solid-solid chemical reaction has never been reported. As much as the chemism is cloudy, the preparation method of solid-solid reaction at room temperature had been seldom studied. In this experiment, the potassium borohydride is thermodynamically unstable and possesses rather reducing activation. The iron, nickel, and lead can be reduced out from their metal salts using the potassium borohydride as reductant. The standard electrode potentials of the corresponding half-reactions are as follows:

(1) $$\begin{array}{c} {\text{H}}_{2}+2{\text{e}}^{-}\to 2{\text{H}}^{-}\;E^{{}^{\circ}}=-2.251\;\text{V}\\ {\text{Fe}}^{3+}+3{\text{e}}^{-}\to \text{Fe}\;E^{{}^{\circ}}=-0.037\;\text{V}\\ {\text{Ni}}^{2+}+2{\text{e}}^{-}\to \text{Ni}\;E^{{}^{\circ}}=-0.246\;\text{V}\\ {\text{Pb}}^{2+}+2{\text{e}}^{-}\to \text{Pb}\;E^{{}^{\circ}}=-0.126\;\text{V} \end{array}$$

Although the above standard electrode potentials of the half-reactions are those in the aqueous solution, the *E*° values may be used as a reference to discuss the room temperature solid-solid reaction. Obviously, the H^−^ anions in the potassium borohydride can very easily reduce the Fe^3+^, Ni^2+^, and Pb^2+^cations to their corresponding atoms. Because the electronegativity of iron (1.8), nickel (1.9) and lead (1.9) is less than that of boron (2.0), the iron, nickel, lead, and boron atoms can form the alloy. In theory, more Fe^3+^ ion should be reduced by KBH_4_ than the Ni^2+^ and Pb^2+^ ions in the experiment. But in fact, the content of iron in the resultants is far less than the amount of iron added in the experiment. Where did the iron go? We observed that the effluent of the washed the resultants was a pale green clear liquid. The pH of the effluent was measured using pH paper, and pH value of the effluent was about 6. This indicated that the effluent did not contain a great deal of the Fe^3+^ ion. In order to further study the reason about the reduction of the iron content in the resultants, we made the qualitative experiment to determine the Fe^2+^ ion in the effluent and the mix solution of the metal salts. The Fe^2+^ ion can form the blood-red complex with phenanthroline. By the addition of phenanthroline, the effluent could become immediately blood-red, but the mix solution of the metal salts did not redden. This illustrated the presence of ferrous ion in the effluent. In order to demonstrate the validity of the conclusion, we used the alkali to form the precipitate and observed the gradual change on the color of the precipitate. When the sodium hydroxide solution was added to the effluent, a large amount of white precipitate was formed in the beginning, and then the precipitate turned gradually into dark green. After standing for some time, the precipitate turned slowly to orange-red. This fits well with the color change process in which the ferrous ion is precipitated under alkaline condition and then oxidized to iron ion by air. The above testing results indicate that a part of iron ion in the experiment is reduced to ferrous ion and is not reduced entirely to iron. The standard electrode potentials of restoring the ferric ion to ferrous ion and restoring ferrous ion to iron in the corresponding half-reactions are listed as follows:

(2) $$\begin{array}{c} {\text{Fe}}^{3+}+{\text{e}}^{-}\to {\text{Fe}}^{2+}\;E^{{}^{\circ}}=0.770\;\text{V}\\ {\text{Fe}}^{2+}+2{\text{e}}^{-}\to \text{Fe}\;E^{{}^{\circ}}=-0.407\;\text{V} \end{array}$$

It is clear that the iron ion is more easily reduced to the ferrous ion. Therefore, the iron ions in the experiment could first be reduced to the ferrous ions, and then the ferrous ions were reduced to iron atoms. However, the reduction of the Fe^2+^ ion to Fe atom is more difficult than the reduction of the Ni^2+^ ion to Ni atom and the Pb^2+^ ion to Pb atom. In this case, many irons ions in the reaction are reduced to the ferrous ions in a short time, but not iron atoms. This also supports the above composition analyses of the resultants. Perhaps, this is why the mole ratio of *n*(Fe) : *n*(Ni) : *n*(Pb) in the resultants is much less than 1.00 : 1.00 : 0.500, which is the mole ratio of *n*(FeCl_3_·6H_2_O) : *n*(NiCl_2_·6H_2_O) : *n*[Pb(CH_3_COO)_2_·3H_2_O] in the reaction raw materials. The mole ratios of *n*(Fe) : *n*(Ni) : *n*(Pb) in the resultants **1**, **2**, and **3** are 0.394 : 1.00 : 0.479, 0.384 : 1.00 : 0.498, and 0.446 : 1.00 : 0.464, respectively. Hence, the percentage content of iron in resultants becomes bigger through increasing potassium borohydride addition. It is feasible that the Fe-Ni-Pb-B alloy nanoparticles are prepared by room temperature solid-solid chemical reaction.

## 4. Conclusions

In summary, the Fe-Ni-Pb-B multicomponent alloy nanoparticles can be prepared very easily by a simple solid-solid chemical reaction method at room temperature. The advantages of this synthetic method are simple and convenient operation, high yield, energy saving, and being environmental friendly; it is in accordance with the requirements of green chemistry. The characterization results indicate that the resultants are mainly consisted of nanoamorphous phase and a minute amount of nanocrystalline phase, and the average diameter is about 25 nm. The resultants are all soft magnetic behavior. The mole ratio of potassium borohydride and the metal salts has an obvious effect on the resultants in composition, crystal structure, and magnetism.

## Figures and Tables

**Table 1:** The testing data of ICP of the resultants.

Resultant	*n* (metal salts) : *n* (KBH_4_)	Fe (%)	Ni (%)	Pb (%)	B (%)	Total (%)
1	1.00 : 1.25	9.45	25.18	42.57	0.19	77.39
2	1.00 : 2.00	9.66	26.45	46.50	0.31	82.92
3	1.00 : 3.00	12.13	28.56	46.79	0.22	87.70

**Table 2:** The saturation magnetization, coercivity, and remanent magnetization of the resultants.

Resultant	Saturation magnetization (emu g^−1^)	Coercivity (Oe)	Remanent magnetization (emu g^−1^)
1	9.01	65.85	1.30
2	17.59	77.85	2.34
3	21.22	54.17	2.20

## References

[1] Wen M, Zhong MF, Wu KJE (2006). Soft magnetic Co–Fe–B–P and Co–Fe–V–B–P amorphous alloy nano-particles prepared by aqueous chemical reduction. *Journal of Alloys and Compounds*.

[2] Lai JKL, Shao YZ, Shek CH, Lin GM, Lan T (2002). Investigation on bulk Nd–Fe–Al amorphous/nano-crystalline alloy. *Journal of Magnetism and Magnetic Materials*.

[3] Lee DS, Jung TK, Kim MS, Kim WY, Yamagata H (2007). Development of nano-structured Al–Si–Fe based bulk alloys from atomized powders. *Materials Science and Engineering A*.

[4] Khajepour M, Sharafi S (2012). Characterization of nanostructured Fe–Co–Si powder alloy. *Powder Technology*.

[5] Suh YJ, Jang HD, Chang H, Kim WB, Kim HC (2006). Size-controlled synthesis of Fe–Ni alloy nanoparticles by hydrogen reduction of metal chlorides. *Powder Technology*.

[6] Wei Z, Li ZR, Jiang Z (2008). In-situ XAFS study on structures and devitrifications of Ni–B nano-amorphous alloys. *Journal of Alloys and Compounds*.

[7] Lu DS, Li WS, Jiang X, Tan CL, Zeng RH (2009). Magnetic field assisted chemical reduction preparation of Co–B alloys as anode materials for alkaline secondary battery. *Journal of Alloys and Compounds*.

[8] Ge Y, Ying G, Bangwei Z, Lingling W, Yifang O, Shuzhi L (1998). Preparation and thermal properties of amorphous Fe–W–B alloy nano-powders. *Journal of Materials Processing Technology*.

[9] Rezvani MR, Shokuhfar A (2012). Synthesis and characterization of nano structured Cu–Al–Mn shape memory alloy by mechanical alloying. *Materials Science and Engineering A*.

[10] Ai FR, Yao AH, Huang WH, Wang DP, Zhang X (2010). Synthesis of PVP-protected NiPd nanoalloys by modified polyol process and their magnetic properties. *Physica E*.

[11] Gurmen S, Guven A, Ebin B, Stopić S, Friedrich B (2009). Synthesis of nano-crystalline spherical cobalt-iron (Co–Fe) alloy particles by ultrasonic spray pyrolysis and hydrogen reduction. *Journal of Alloys and Compounds*.

[12] Fan ZG, Li CY (2007). Preparation of NiB alloy from spent NiAl catalysts by induction furnace. *Journal of Alloys and Compounds*.

[13] Yuan ML, Tao JH, Yu L (2011). Synthesis and magnetic properties of Fe–Ni alloy nanoparticles obtained by hydrothermal reaction. *Advanced Materials Research*.

[14] Chen CM, Jehng JM (2004). Amination application over nano-Mg–Ni hydrogen storage alloy catalysts. *Applied Catalysis A*.

[15] Yedra A, Barquín LF, Sal JCG, Pankhurst QA (2003). Nanoscale alloys prepared by sodium borohydride reduction of aqueous Fe–Cu and Co–Cu solutions. *Journal of Magnetism and Magnetic Materials*.

[16] de Resende VG, de Grave E, da Costa GM, Janssens J (2007). Influence of the borohydride concentration on the composition of the amorphous Fe–B alloy produced by chemical reduction of synthetic, nano-sized iron oxide particles—part II: goethite. *Journal of Alloys and Compounds*.

[17] Aonuma M, Ogawa H, Tamai Y Process for production of ferromagnetic powder.

[18] Zhong GQ, Zhou HL, Jia YQ (2013). A simple method for preparation of copper nanocrystalline particles. *Nanoscience and Nanotechnology Letters*.

[19] Zhong GQ, Zhou HL, Zhang JR, Jia YQ (2005). A simple method for preparation of Bi and Sb metal nanocrystalline particles. *Materials Letters*.

[20] Zhong GQ, Zhou HL, Jia YQ (2008). Preparation of amorphous Ni–B alloys nanoparticles by room temperature solid-solid reaction. *Journal of Alloys and Compounds*.

[21] Liu ML, Zhou HL, Chen YR, Jia YQ (2005). Room temperature solid-solid reaction preparation of iron-boron alloy nanoparticles and Mössbauer spectra. *Materials Chemistry and Physics*.

[22] Saatçi B, Maraşlı N, Gündüz M (2007). Thermal conductivities of solid and liquid phases in Pb–Cd and Sn–Zn binary eutectic alloys. *Thermochimica Acta*.

[23] El-Danaf EA, Khalil KA, Soliman MS (2012). Effect of equal-channel angular pressing on superplastic behavior of eutectic Pb–Sn alloy. *Materials and Design*.

[24] Xie H, Yang GC, La PQ (2004). Microstructure and wear performance of Ni–20 wt.% Pb hypomonotectic alloys. *Materials Characterization*.

[25] Silva AP, Garcia A, Spinelli JE (2011). Microstructure morphologies during the transient solidification of hypomonotectic and monotectic Al–Pb alloys. *Journal of Alloys and Compounds*.

[26] Cui HB, Guo JJ, Su YQ (2007). Effect of Cr addition on microstructure and wear resistance of hypomonotectic Cu–Pb alloy. *Materials Science and Engineering A*.

[27] Xie H, Sun J, Yang GC, Chen YZ, Lu ZL (2007). Liquid phase separation and its thermodynamic description in undercooled Ni–Pb hypermonotectic melts. *Materials Science and Engineering A*.

[28] Monchoux JP, Rabkin E (2002). Microstucture evolution and interfacial properties in the Fe–Pb system. *Acta Materialia*.

[29] Hsu JH, Huang YH (1999). Tunneling magnetoresistance effect in Fe–Pb–O and Fe–PbO granular films: a comparison. *Journal of Magnetism and Magnetic Materials*.

[30] Fang F, Zhu M, Deng HQ, Shu XL, Hu WY (2004). Self-diffusion of Al and Pb atoms in Al–Pb immiscible alloy system. *Materials Science and Engineering B*.

